# Cortical network underlying audiovisual semantic integration and modulation of attention: An fMRI and graph-based study

**DOI:** 10.1371/journal.pone.0221185

**Published:** 2019-08-23

**Authors:** Yang Xi, Qi Li, Ning Gao, Siyuan He, Xiaoyu Tang

**Affiliations:** 1 School of Computer Science and Technology, Changchun University of Science and Technology, Changchun, Jilin, P.R. China; 2 School of Computer Science, Northeast Electric Power University, Jilin, Jilin, P.R. China; 3 School of Psychology, Liaoning Normal University, Dalian, Liaoning, P.R. China; Banner Alzheimer’s Institute, UNITED STATES

## Abstract

Many neuroimaging and electrophysiology studies have suggested that semantic integration as a high-level cognitive process involves various cortical regions and is modulated by attention. However, the cortical network specific to semantic integration and the modulatory mechanism of attention remain unclear. Here, we designed an fMRI experiment using “bimodal stimulus” to extract information regarding the cortical activation related to the effects of semantic integration with and without attention, and then analyzed the characteristics of the cortical network and the modulating effect of attention on semantic integration. To further investigate the related cortical regions, we constructed a functional brain network for processing attended AV stimuli to evaluate the nodal properties using a graph-based method. The results of the fMRI and graph-based analyses showed that the semantic integration with attention activated the anterior temporal lobe (ATL), temporoparietal junction (TPJ), and frontoparietal cortex, with the ATL showing the highest nodal degree and efficiency; in contrast, semantic integration without attention involved a relatively small cortical network, including the posterior superior temporal gyrus (STG), Heschl’s gyrus (HG), and precentral gyrus. These results indicated that semantic integration is a complex cognitive process that occurs not only in the attended condition but also in the unattended condition, and that attention could modulate the distribution of cortical networks related to semantic integration. We suggest that semantic integration with attention is a conscious process and needs a wide cortical network working together, in which the ATL plays the role of a central hub; in contrast, semantic integration without attention is a pre-attentive process and involves a relatively smaller cortical network, in which the HG may play an important role. Our study will provide valuable insights into semantic integration and will be useful for investigations on multisensory integration and attention mechanism at multiple processing stages and levels within the cortical hierarchy.

## Introduction

The integration of information from multiple senses is a fundamental requirement for recognition of the world around us. Multisensory inputs are integrated across different stages of stimulus processing and can be modulated by attention [1–2]. Behavioral data have shown that temporally, spatially, and semantically congruent information has a facilitatory effect on performance such that bimodal stimuli are detected and discriminated faster or more accurately [3–4]. The facilitatory effect of spatial and temporal congruence (or approximate congruence) has been considered to be due to the early neural integration stages [5]. For example, it has been demonstrated in the superior colliculus of cats, termed as sensory integration [5–6]. However, this early signal-statistic-dependent sensory integration cannot account for the behavioral consequences of semantic congruency, which are termed as semantic integration [6].

Semantic integration of multisensory information is an essential cognitive process for recognizing objects and communicating effectively and has been widely studied using functional magnetic resonance imaging (fMRI) [2, 7–9]. Neuroimaging studies have shown that the integration of multisensory inputs containing semantic information involves multiple cortical regions [8–11]. For example, Ye *et al*. showed that both superior temporal regions plus the medial prefrontal cortex are involved in the integration of speech and lip movements [8]. Similarly, significant activations at the right middle and superior temporal gyri were found when localization of sound sources was semantically congruent with visual stimuli [9]. In addition, Beauchamp *et al*. found that the posterior superior temporal sulcus and middle temporal gyrus responded more strongly to semantic audiovisual stimuli than to either auditory or visual stimuli [12]. Moreover, the temporoparietal junction (TPJ), which is composed of the posterior temporal lobe and the inferior parietal lobule [13–15], and the anterior temporal lobe (ATL) [13–18] have been widely considered to play a key and special role in cross-modal semantic representation [13–18]. We speculate that the semantic information interacts within multiple cortical regions, some of which may act as hubs forming a cortical network related to the stage of semantic integration.

Activation of the cortical network may vary across different stages of multisensory integration and may be affected by attention [19]. Brains often selectively attend to some stimuli while ignoring the others, and the attended stimulus is believed to be selectively processed [20]. Selection of attention can occur in a top-down manner, which is based on the intentions and task of the observer, and can also occur in a bottom-up manner, in which attention can shift without voluntary control [1]. Neuroimaging studies have demonstrated that the brain selectively integrates the semantic information from attended objects [20] and that multisensory integration requires the objects to be fully attended [1]. In contrast, many electrophysiological studies have explored the modulation of attention on late ERP (event-related potential) components related to semantic processing and suggested that cross-modal semantic processing occurs even when the semantic information is not attended [21]. These electrophysiological studies have done much excellent work on the interaction between attention and cross-modal semantic processing, but these results cannot identify the cortical regions associated with modulation of attention, due to the low spatial resolution of the ERP method.

To date, the cortical network related to the effects of semantic integration and modulation of attention remains unclear. Many neuroimaging studies have investigated the cortical network related to semantic integration by comparing the activation related to processing semantically congruent and incongruent audiovisual stimuli [22–23]. However, it has been argued that incongruency manipulations violate natural multisensory relationships and invoke error detection processes, and their role in the characterization of natural multisensory integration processes may be limited [2]. A more direct approach is to remove other integration effects to obtain only the effects related to semantic integration.

In the present study, we removed the effects related to early sensory integration and extracted the effect of semantic integration by utilizing “bimodal stimulus” based on a previous study [2], and then we explored the cortical network specific to the semantic integration. By comparing the activations related to the effects of semantic integration with and without attention, we discerned the modulation of attention. In addition, to further identify which region activated in semantic integration with attention is the central hub, we constructed a functional brain network for semantically congruent audiovisual stimuli presented on the attended side and analyzed the nodal properties of degree and efficiency. Our study will provide valuable insights into the stage of semantic integration, and this is important for understanding multisensory integration [24–26].

## Methods

### Participants

Eighteen healthy volunteers (9 females; age range: 21–27 years; mean age: 24 years) participated in this fMRI study. All participants had normal or corrected-to-normal vision and normal hearing capability; were neurologically healthy; did not have a history or diagnosis of mental illness; did not use psychoactive medication or drugs; and did not have any permanent metal in their body. Participants were compensated with ¥100/h for the fMRI experiments. The experimental protocol was approved by the Ethics Committee of Changchun University of Science and Technology. After receiving a full explanation of the purpose and risks of the study, all participants gave written informed consent for all experiments as per the protocol approved by the institutional research review board. All procedures were carried out in accordance with the approved guidelines.

### Stimuli

**Fig 1:**
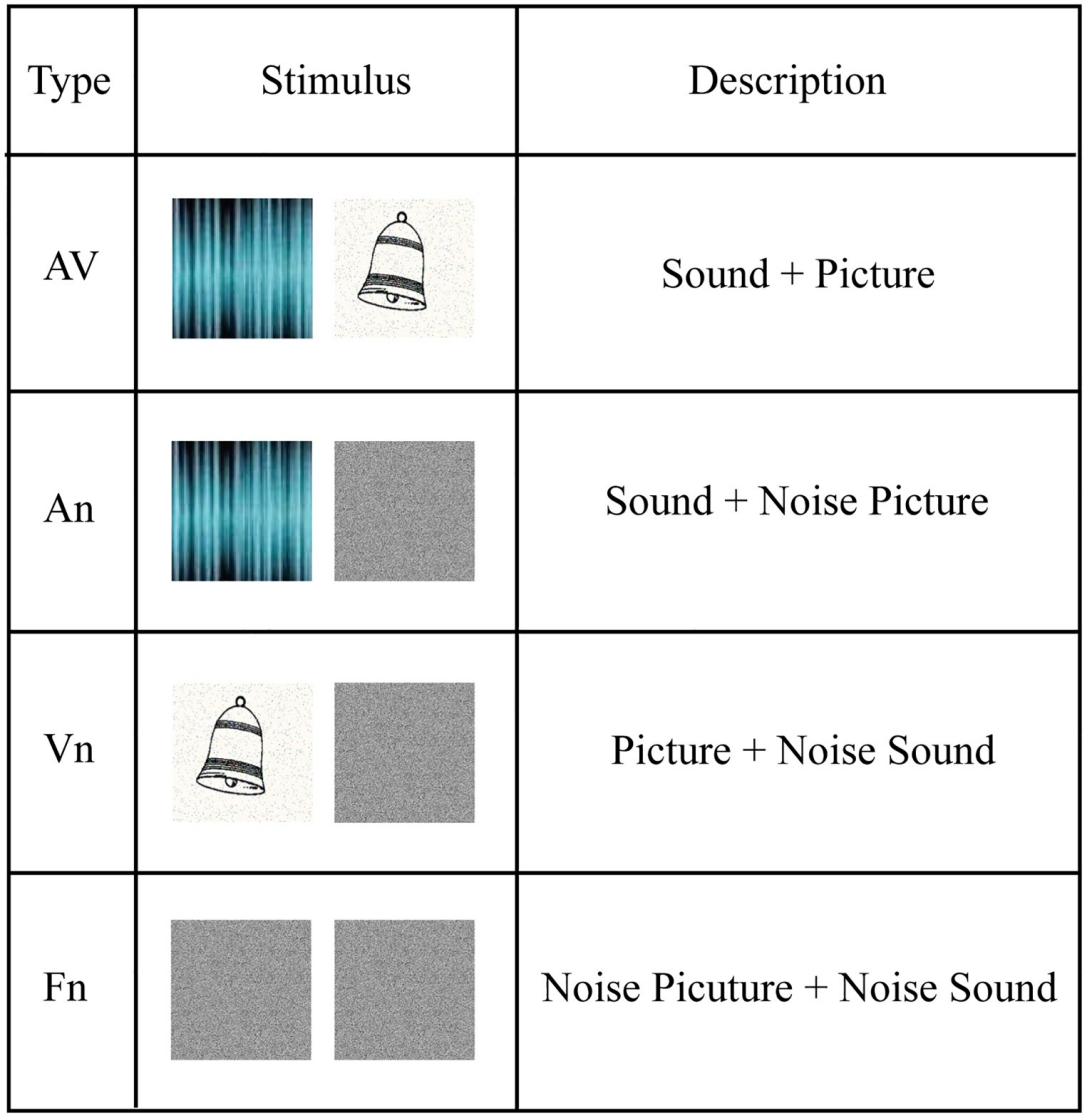
Four types of bimodal stimuli and examples. AV stimuli consist of semantically congruent picture and sound; An stimuli consist of a sound and a noise picture; Vn stimuli consist of a picture and a noise sound; Fn stimuli consist of noise picture and sound.

A “bimodal stimulus” was constructed by using a visual or auditory noise presented simultaneously to obtain four groups: bimodal visual (Vn), bimodal auditory (An), bimodal empty (Fn), and bimodal audiovisual (AV) stimuli. Vn stimuli consisted of an auditory white noise and a visual stimulus presented simultaneously, while An stimuli consisted of a visual white noise and an auditory stimulus presented simultaneously. Fn stimuli consisted of auditory and visual white noise presented simultaneously, while AV stimuli consisted of semantically congruent auditory and visual stimuli presented simultaneously (Fig 1). Inanimate objects and animals were used as stimulus materials, since they often have distinct visual and auditory features, providing ideal stimulus sets for examining this integration process [2, 12]. Visual stimuli were gray-scale pictures of the inanimate objects (e.g., bell, guitar, car, and clock) or animals (e.g., cat, dog, cow, and frog) from the international common Snodgrass-Vanderwart white-black line graphic library and were processed using Adobe Illustrator software (Adobe Systems Inc., SAN Jose, California, USA). Finally, auditory stimuli were the sounds corresponding to the inanimate objects or animals obtained from the Internet and were processed using CoolEditPro 2.1 software (Adobe Systems Inc., SAN Jose, California, USA). Auditory noise was a white noise sound with identical length as auditory stimuli, and visual noise was a white noise image with identical size as visual stimuli. Stimulus presentation was controlled by a personal computer running Presentation 0.71 software (Neurobehavioral Systems Inc., Albany, California, USA).

The visual stimuli and visual noise (6.0 cm × 4.8 cm, subtending a visual angle of approximately 4.3°) were projected onto a screen placed behind the head of the participants at the end of the scanner bore, visible to participants by a mirror placed within the MR head coil. All the visual stimuli were presented on the left or right side of the display at an angle of approximately 6° from a centrally presented fixation point located directly in front of the participants’ eyes, with a duration of 300 ms. The auditory stimuli and auditory noise were presented through earphones (44 kHz sampling rate, duration 400 ms, 10 ms rise and fall periods). The hearing threshold of each participant was measured before the recordings, and the volume of the stimuli was set to 80 dB [2]. In case the participant requested, volume was increased. The interstimulus interval (ISI) varied randomly among 2 s, 4 s and 6 s. To maintain participants’ attention during the discrimination task, both auditory and visual components were presented in a degraded manner [2, 27]. The visual stimuli were degraded by random noise images of the identical size. Similar to the procedure for degrading visual images, auditory stimuli were degraded by a random noise sound of identical length. The item-specific degradation level was determined based on a behavioral test before the semantic discrimination task to obtain an across-participant accuracy of 85% averaged over all items.

### Procedure

**Fig 2:**
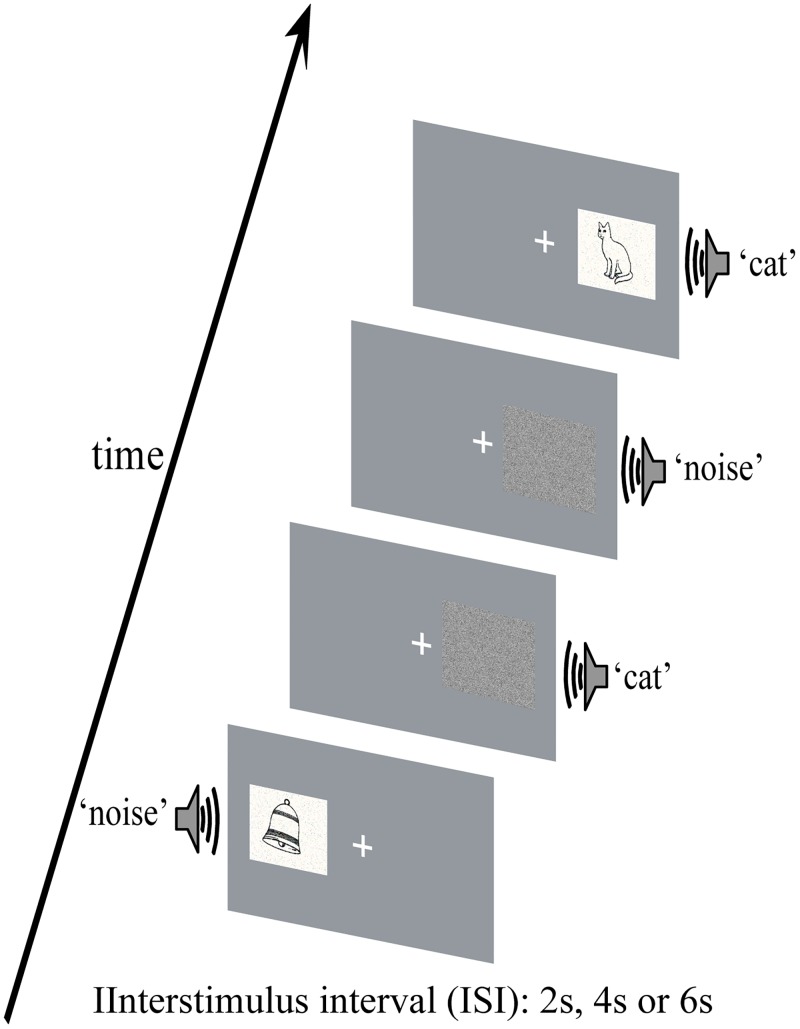
Schematic of the experimental paradigm. The interstimulus interval (ISI) is 2, 4 or 6 seconds.

An event-related fMRI design was adopted. Participants continued training until the experimenter was convinced that they understood the task. Eight sessions had to be completed by each participant, and each session consisted of 40 An stimuli, 40 Vn stimuli, 40 AV stimuli, and 40 Fn stimuli, as shown in Fig 2. The frequencies of the inanimate object and animal stimuli were both 50% for each group of An, Vn and AV stimuli. There were seven types of stimuli (2 (inanimate object and animal) × 3 (An, Vn, and AV) + 1 (Fn)), which were presented with equal probability on the left and right sides of the participant according to a pseudorandom sequence.

During the experiment, participants were instructed to minimize blinking and bodily movements to avoid movement artifacts and were required to fix their eyes on a centrally presented fixation point and to attend to the stimuli on one side while ignoring the stimuli on the opposite side. The task was to press the left button when hearing or /and seeing the animal stimuli and press the right button when hearing or /and seeing the inanimate object stimuli on attended side, with responding as quickly and accurately as possible. Participants were required to attend to the left side in four of eight sessions (named as left session), and to attend to the right side in the other four sessions (named as right session). The two types of sessions were conducted in an alternating fashion. All participants were allowed to take a 5 min break between sessions.

### Acquisition of fMRI data

A 3T fMRI scanner (Siemens) at the Sino Japanese Friendship Hospital of Jilin University was used to acquire both T1-weighted anatomical images (repetition time (TR) = 8600 ms; echo time (TE) = 4 ms; field-of-view (FOV) = 192 mm; flip angle (FA) = 90°; 128 slices; voxel size = 1×1×1 mm) and T2-weighted gradient echo planar imaging sequence (TR = 2 s; TE = 30 ms; FOV = 192 mm; FA = 90°; 33 slices; voxel size = 3×3×3 mm). There were eight sessions with a total of 160 volume images per session. The high-resolution anatomical image volume was acquired at the end of the experiment.

### Data analysis

#### Behavioral data analysis

Reaction times (RTs) and hit rates (HRs) were computed separately for each type of stimulus (An, Vn and AV) and side (left and right), and analyzed using a repeated-measures analysis of variance (ANOVA) with type (An, Vn, and AV) and side (left and right) as subject factors. All statistical analysis was performed using SPSS software (version 22, IBM Inc., UAS). The α level was set to 5%.

#### fMRI data analysis

The processing of each type of stimulus (An, Vn, AV and Fn) is described in Table 1. The effect of early sensory integration can be removed by comparing the audiovisual interaction in AV+Fn condition relative to those in the Vn+An condition, allowing one to extract the effect involved in the semantic integration. For all left sessions, the comparison of (AV+Fn) and (Vn+An) presented on left side reflected the effect of semantic integration with attention, while the comparison of that presented on right side reflected the effect of semantic integration without attention. The situations were similar for all right sessions. The regions associated with the modulation effect of attention were identified by comparing the semantic integration with and without attention.

**Table 1:** The processing of An, Vn, AV, and Fn stimuli in the brain.

	AV	Vn	An	Fn	(AV+Fn)-(An+Vn)
**Visual stimulus**	√	√			
**Auditory stimulus**	√		√		
**Visual noise**			√	√	
**Auditory noise**		√		√	
**Sensory integration**	√	√	√	√	
**Semantic integration**	√				√

The fMRI imaging data were analyzed using the SPM12 software package (Wellcome Department of Cognitive Neurology, London, UK) running under Matlab2012a (MathWorks Inc., Natick, Massachusetts, USA). Six scans at the beginning of the measurement were removed automatically from the data set. Functional data were slice time-corrected, motion-corrected, normalized into standard stereotactic space using the Montreal Neurological Institute (MNI) template, and smoothed using a 6.0-mm full-width half-maximum Gaussian kernel. To reduce motion-related artifacts, session-specific realignment parameters from preprocessing were used as first-level covariates. Statistical analysis was performed at the individual participant level by using the general linear model framework, and the blood oxygen level-dependent response was modeled as the neural activity convolved with a canonical hemodynamic response function. The contrast of (AV+Fn > An+Vn) with attention and (AV+Fn > An+Vn) without attention in all left-sessions and right-sessions were implemented. All individual functional localization data were then used for the group-level statistics. One-sample t-tests were used to construct statistical parametric maps at the group level for (AV+Fn > An+Vn) contrasts, determining the voxels in which activity differed significantly from zero, i.e., the voxels that showed significant activity in the processes of audiovisual semantic integration with and without attention.

### Graph-based analysis for the nodal properties of the functional brain network

The fMRI data corresponding to the AV stimuli presented on the attended side was entered into the CONN toolbox to construct a functional brain network [28–30]. The anatomical volumes were segmented into gray matter, white matter, and CSF areas, and the resulting masks were eroded (one voxel erosion, isotropic 2-mm voxel size) to minimize partial volume effects. The temporal time series characterizing the estimated subject motion (three-rotation and three-translation parameters, plus another six parameters representing their first-order temporal derivatives), as well as the BOLD time series within the subject-specific white matter mask (three PCA parameters) and CSF mask (three PCA parameters), were used as temporal covariates and removed from the BOLD functional data by using linear regression [28]. A network comprises nodes and edges connecting the nodes. In the present study, we defined cortical regions of interest (ROIs) from HOA112 atlas as nodes, which parcellated the brain into 112 ROIs. The mean time series for each ROI was extracted from the preprocessed images, and Pearson correlation was applied to the mean time series as the task-dependent functional connectivity between nodes. First-level (within-subjects) connectivity analysis was performed across all sessions. After computation of individual ROI-to-ROI connectivity matrices, the measure was then entered into a second-level general linear model to obtain population-level estimates and inferences. False-positive control in ROI-to-ROI analysis was implemented by using false discovery rate (FDR)-corrected p-values [28].

We entered the functional brain network into the GRETNA toolbox to perform the graph-based analysis. The graphs for ROIs and their functional connectivity were analyzed on an individual-subject basis. We selected the equal-interval sparsity threshold range (ranging from 0.05 to 0.5 with a partition interval of 0.05), and the average values of area under curve (AUC) were used for statistical analysis in order to provide a scalar that did not depend on the specific threshold selection. We computed the nodal degree and nodal efficiency, and identified the nodes with degree and efficiency values larger than the sum of the average values and the standard deviation across all nodes of the network as a hub node. In addition, we further calculated the connectivity pattern between the hub with highest nodal degree and nodal efficiency and other regions.

## Results

### Behavioral results

A repeated-measures ANOVA for the two factors modality (An, Vn, and AV) and hemisphere (left and right) showed no significant main effects of hemisphere in the RTs [F_1,18_ = 0.059, p = 0.811] or HRs [F_1,18_ = 1.759, p = 0.178]. Therefore, the RTs and HRs from the left and right hemispheres were combined to improve the signal-noise ratio. Mean RTs and HRs for An, Vn, and AV stimuli are shown in Table 2.

**Table 2:** Mean response times (RTs) and hit rates (HRs). Response times are presented in milliseconds (ms). Hit rates are represented as percentage values.

RTs (ms)		HRs (%)	
**An**	816.32 (99.62)	**An**	96.28(5.38)
**Vn**	735.39 (92.95)	**Vn**	91.92 (8.98)
**AV**	731.42 (88.86)	**AV**	98.25 (2.59)

**Fig 3:**
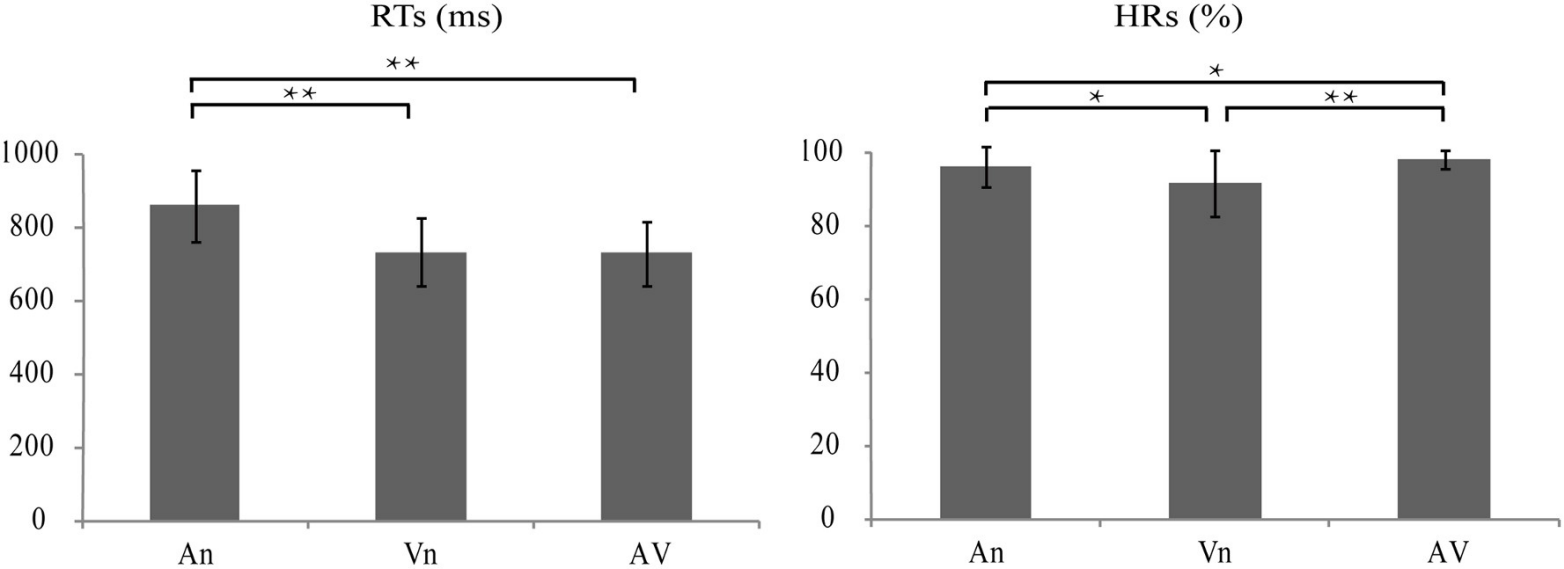
Bar plots showing response times (RTs) and hit rates (HRs) for the target under the three conditions (An, Vn, and AV stimuli). Left: RTs (across participants, mean ± SD). Right: HRs (across participants, mean ± SD). **Significant at p < 0.0005, *Significant at p < 0.05.

The results of RT analysis revealed main effects of modality [F_2,74_ = 16.774, p < 0.0005], indicating that RTs significantly differed among modalities. *Post hoc* comparisons revealed that RTs to AV stimuli were significantly faster than those to An stimuli [t_37_ = 18.219, p < 0.0005], and they were not significantly faster than those to Vn stimuli [t_37_ = 0.501, p = 0.619]. RTs to Vn stimuli were also faster than those to An stimuli [t_37_ = 13.943, p < 0.0005] (Fig 3). The result of HRs analysis revealed main effects of modality [F_2,74_ = 14.485, p < 0.0005]. *Post hoc* comparisons revealed that HRs for AV stimuli were much higher than those for An stimuli [t_37_ = 3.529, p < 0.001] and Vn stimuli [t_37_ = 5.565, p < 0.0005]. HRs for An stimuli were also significantly higher than those for Vn stimuli [t_37_ = 3.787, p < 0.001] (Fig 3).

### fMRI results

To identify the regions related to semantic integration with attention, we implemented the contrast of (AV+Fn) versus (An+Vn) presented on the attended side. Similarly, we obtained the effect of semantic integration without attention by implementing the contrast of (AV+Fn) versus (An+Vn) presented on the unattended side. Tables 3 and 4 show the significantly activated clusters related to semantic integration with and without attention.

**Table 3:** Activated clusters related to semantic integration with attention.

Brain region	Cluster size	Peak T	MNI coordinates (x, y, z)			Brodmman area
Temporoparietal junction: Left						
Angular Gyrus	179	7.08	-48	-70	35	BA39
Supramarginal Gyrus		5.21	-54	-64	29	BA 40
Middle Temporal Gyrus		5.07	-39	-58	23	BA 21
Superior Temporal Gyrus		4.30	-41	-59	20	BA 22
Temporoparietal junction: Right						
Angular Gyrus	128	5.75	51	-70	32	BA 39
Supramarginal Gyrus		4.51	51	-55	29	BA 40
Supramarginal Gyrus		4.49	57	-55	23	BA 40
Middle Temporal Gyrus		4.76	53	-63	26	BA 21
Superior Temporal Gyrus		4.53	56	-61	26	BA 22
Anterior temporal lobe: Left						
Middle Temporal Gyrus	51	4.78	-48	8	-37	BA 21
Middle Temporal Gyrus		4.61	-42	14	-40	BA 21
Middle Temporal Gyrus		4.39	-39	5	-40	BA 21
Middle Temporal Gyrus	37	4.56	-57	-7	-19	BA 21
Middle Temporal Gyrus		4.21	-60	-16	-13	BA 21
Anterior temporal lobe: Right						
Middle Temporal Gyrus	101	5.10	48	8	-34	BA 21
Middle Temporal Gyrus		4.77	54	-4	-25	BA 21
Middle Temporal Gyrus		4.48	54	5	-25	BA 21
Superior Temporal Gyru		4.51	45	11	-31	BA 22
Frontal cortex						
Medial Superior Frontal Gyrus	367	7.06	9	53	44	BA 8
Medial Frontal Gyrus		5.38	15	44	53	BA 8/9
Medial Frontal Gyrus		5.36	-12	38	56	BA 8/9
Superior Frontal Gyrus		4.45	-12	48	41	BA 4/6/8
Medial Superior Frontal Gyrus	132	6.37	-12	47	-16	BA 8
Medial Orbitofrontal cortex		4.63	0	35	-13	BA 10/11
Inferior Frontal Gyrus		4.70	3	44	-16	BA 11
Parietal cortex						
Precuneus	35	6.29	-24	-85	38	BA 7
Precuneus		4.91	-24	-82	41	BA 7
Posterior Cingulate Gyrus		4.67	-6	-52	26	BA 31
Lateral Occipital gyrus	43	5.35	30	-82	38	BA 19
Superior parietal lobule		4.49	30	-76	50	BA 5/7
Precuneus		4.08	21	-82	44	BA 7
Posterior Cingulate Gyrus	144	5.28	9	-49	26	BA 31

*P*_uncorr_ < 0.0005; cluster voxels ≥ 30 voxels.

**Table 4:** Activated clusters related to semantic integration with attention.

Brain region	Cluster size	Peak T	MNI coordinates (x, y, z)			Brodmman area
Precentral Gyrus	90	5.25	-36	-19	59	BA 4
Precentral Gyrus		4.90	-30	-25	65	BA 4
Precentral Gyrus		4.02	-24	-25	59	BA 4
Supramarginal Gyrus	87	4.81	-51	-28	14	BA 40
Heschl’s Gyrus		4.58	-45	-25	8	BA 41
Superior Temporal Gyrus		4.42	-48	-25	8	BA 22
Heschl’s Gyrus	31	4.36	48	-22	11	BA 41
Superior Temporal Gyrus		4.01	48	-21	8	BA 22

*P*_uncorr_ < 0.0005; cluster voxels ≥ 30 voxels.

**Fig 4:**
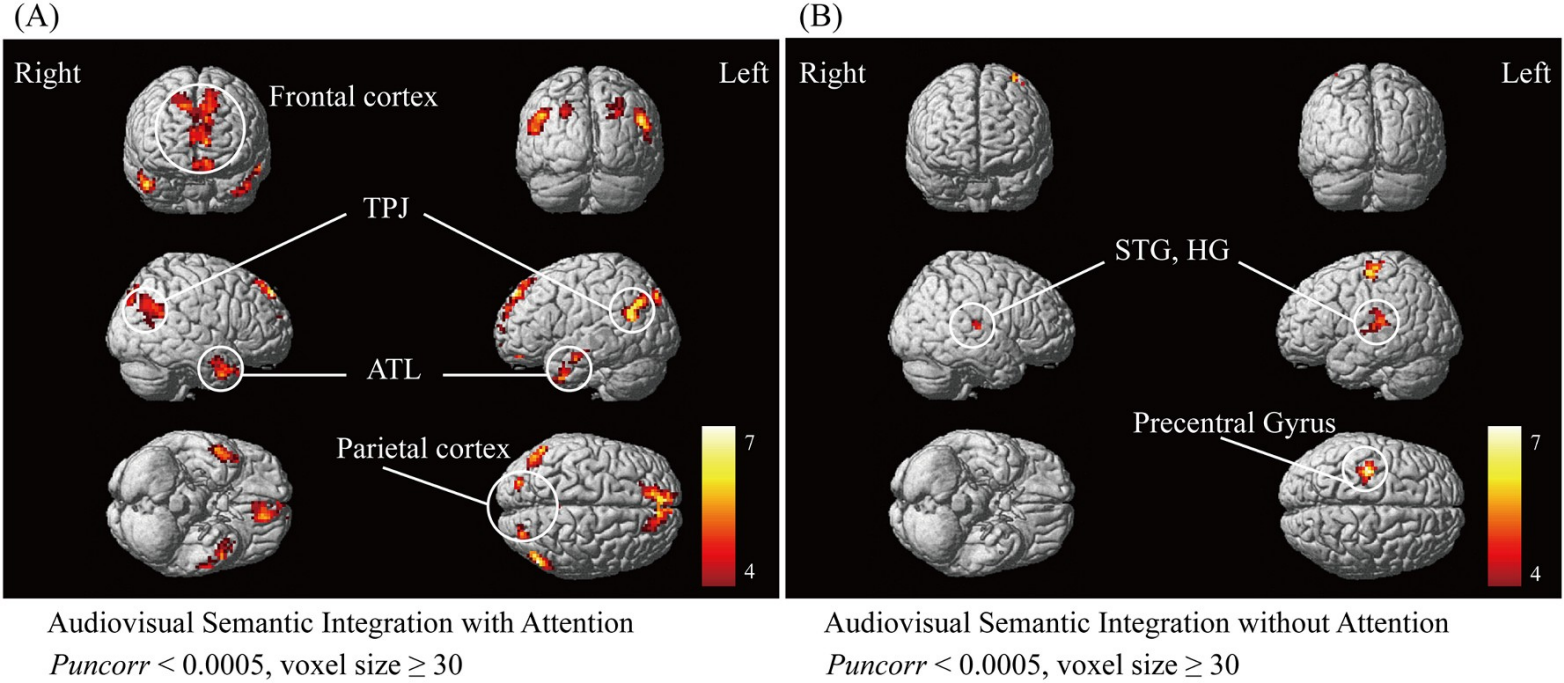
**(A) The fMRI activation observed in contrast of (AV+Fn) > (An+Vn) presented on the attended side, reflecting the cortical network of semantic integration with attention. (B) The fMRI activation observed in contrast of (AV+Fn) > (An+Vn) presented on the unattended side, reflecting the cortical network of semantic integration without attention**. *Puncorr* < 0.0005, cluster voxels ≥ 30 voxels. TPJ, temporoparietal junction; ATL, anterior temporal lobe; STG, superior temporal gyrus; HG, Heschl’s gyru. The color bar indicates T values.

The effect of semantic integration with attention was located in the bilateral temporoparietal junction (TPJ), anterior temporal lobe (ATL), frontal lobe, and parietal lobe cortices. Specifically, the posterior middle temporal gyrus, posterior superior temporal gyrus, angular gyrus, supramarginal gyrus in the TPJ, anterior middle temporal gyrus, anterior superior temporal gyrus in the ATL, medial frontal gyrus, superior frontal gyrus, inferior frontal gyrus and orbitofrontal cortex in the frontal lobe cortex, precuneus, and the posterior cingulate gyrus and superior parietal lobule in the parietal lobe cortex were strongly activated, as shown in Fig 4(A). The activation results indicated that the cortical network related to semantic integration with attention is widely distributed in the prefrontal, parietal and temporal lobes. In contrast, the significant activation corresponding to semantic integration without attention involved a relatively small cortical network, mainly including the posterior superior temporal gyrus (STG), Heschl’s gyrus (HG), and precentral gyrus, as shown in Fig 4(B).

### Nodal degree and efficiency

**Fig 5:**
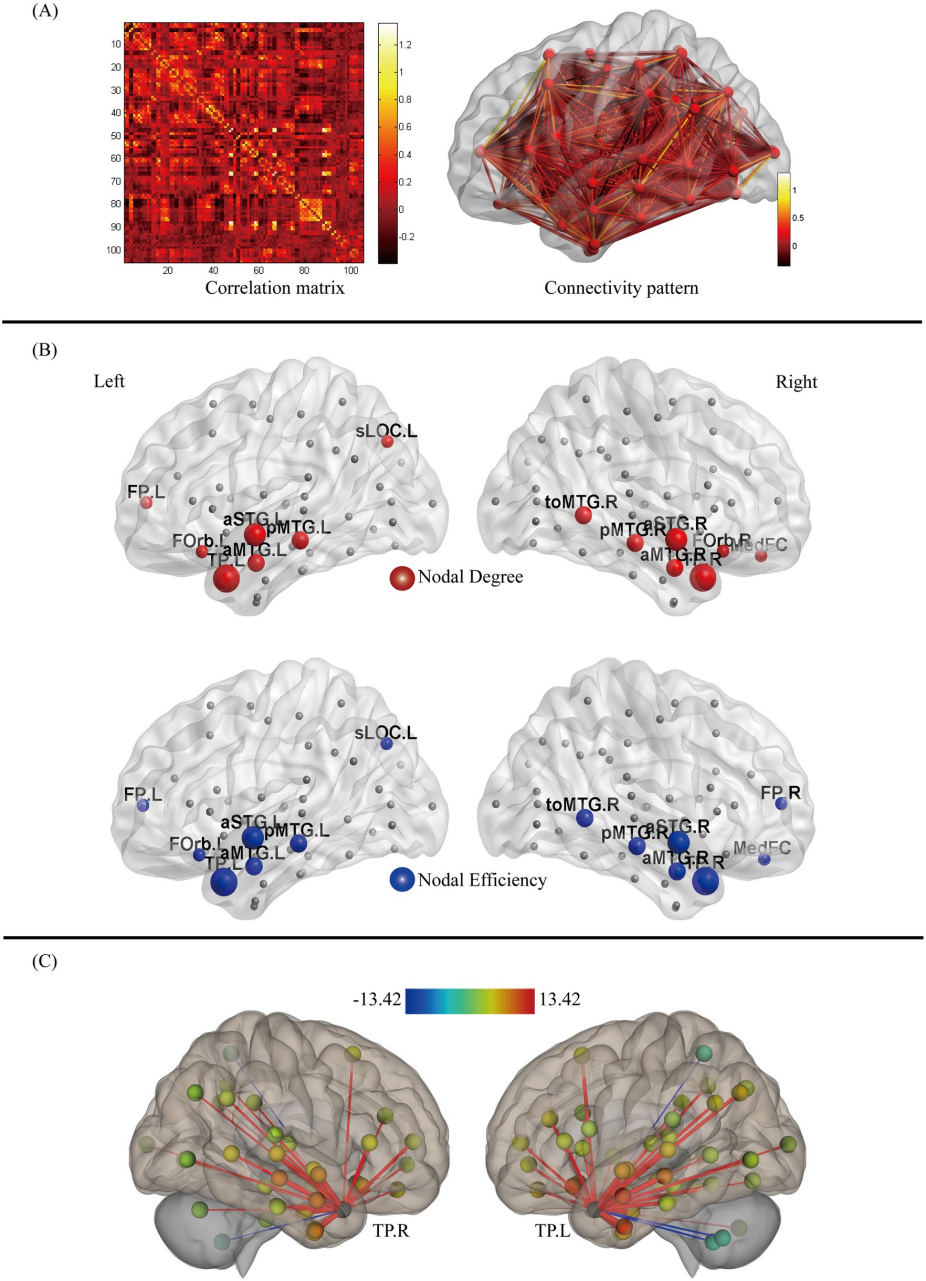
**(A) The correlation matrix and the functional connectivity pattern in the brain for processing AV stimuli attended**. The color bar indicates the strength of connectivity. **(B) The distribution of hubs with high degree and nodal efficiency**. The node that degree or efficiency value is larger than the sum of the average value and the standard deviation across all nodes of the network is defined as a hub node. The red spheres are the hubs with high nodal degree, and the blue spheres are the hubs with high nodal efficiency. The size of the spheres indicates the relative size of the nodal degree or efficiency. **(C) The strength of functional connectivity between bilateral temporal poles (TP.L and TP.R) with other regions in temporal lobes**. The color bar indicates the strength of connectivity.

By constructing the functional brain network, we obtained the correlation matrix and the functional connectivity pattern, as shown in Fig 5(A). Then we computed the nodal degree and efficiency and obtained the hubs, values of which were shown in Table 5.

**Table 5:** The information of hubs with high nodal degree and nodal efficiency.

Node	Description	Degree	Node	Description	Efficiency
TP.L	left temporal pole	17.607	TP.L	left temporal pole	0.2909
TP.R	right temporal pole	17.144	TP.R	right temporal pole	0.2878
aSTG.L	left anterior superior temporal gyrus	16.641	aSTG.L	left anterior superior temporal gyrus	0.2851
aSTG.R	right anterior superior temporal gyrus	16.354	aSTG.R	right anterior superior temporal gyrus	0.2827
pMTG.L	left posterior middle temporal gyrus	16.146	pMTG.L	left posterior middle temporal gyrus	0.2822
pMTG.R	right posterior middle temporal gyrus	15.964	pMTG.R	right posterior middle temporal gyrus	0.2812
toMTG.R	right temporo-occipital middle temporal gyrus	15.880	toMTG.R	right temporo-occipital middle temporal gyrus	0.2806
aMTG.L	left anterior middle temporal gyrus	15.660	aMTG.L	left anterior middle temporal gyrus	0.2794
aMTG.R	right anterior middle temporal gyrus	15.651	aMTG.R	right anterior middle temporal gyrus	0.2785
FOrb.L	left frontal orbital cortex	15.481	sLOC.L	left lateral occipital cortex	0.2775
sLOC.L	left lateral occipital cortex	15.352	FOrb.L	left frontal orbital cortex	0.2773
MedFC	frontal medial cortex	15.087	FP.L	left frontal pole	0.2746
FP.L	left frontal pole	14.964	MedFC	frontal medial cortex	0.2737
FOrb.R	right frontal orbital cortex	14.799	FP.R	right frontal pole	0.2736

The distribution of hubs with high nodal degree and nodal efficiency is shown in Fig 5(B), and the values of nodal degree and efficiency of hubs are shown in Table 5. The result showed that the fourteen top degree nodes were mainly concentrated in the temporal and frontal lobes, including the left temporal pole (TP.L), right temporal pole (TP.R), left anterior superior temporal gyrus (aSTG.L), right anterior superior temporal gyrus (aSTG.R), left posterior middle temporal gyrus (pMTG.L), right posterior middle temporal gyrus (pMTG.R), right temporooccipital middle temporal gyrus (toMTG.R), left anterior middle temporal gyrus (aMTG.L), the right anterior middle temporal gyrus (aMTG.R), left frontal pole (FP.L), left frontal orbital cortex (FOrb.L), right frontal orbital cortex (FOrb.R), frontal medial cortex (MedFC), and left superior lateral occipital cortex (sLOC.L). Interestingly, as shown in Fig 5(B), the hubs with high efficiency were almost identical to those with high degree, including left temporal pole (TP.L), right temporal pole (TP.R), left anterior superior temporal gyrus (aSTG.L), right anterior superior temporal gyrus (aSTG.R), left posterior middle temporal gyrus (pMTG.L), right posterior middle temporal gyrus (pMTG.R), right temporo-occipital middle temporal gyrus (toMTG.R), left anterior middle temporal gyrus (aMTG.L), right anterior middle temporal gyrus (aMTG.R), left superior lateral occipital cortex (sLOC.L), left frontal orbital cortex (FOrb.L), left frontal pole (FP.L), frontal medial cortex (MedFC), right frontal pole (FP.R). In addition, the values of nodal degree and efficiency of these hubs decreased gradually from the anterior temporal lobe to the posterior temporal lobe. Most importantly, the distribution showed that the two nodes with the highest degree and efficiency were both TP.L and TP.R. Further analysis showed that the connectivity strength between TP in the left and right hemispheres was the highest, followed by the connectivity between TP and other regions in the temporal lobe, including aSTG.R, aSTG.L, aMTG.R, aMTG.L, pMTG.R, pMTG.L, aITG.R, and aITG.L, and the connectivity strength between the TP and other regions in the temporal lobe decreased gradually from the anterior to the posterior temporal lobe, as shown in Fig 5(C).

## Discussion

The present study aimed to identify the cortical networks underlying audiovisual semantic integration and explore the modulatory mechanism of attention. Behavioral evidence for audiovisual semantic integration was obtained from HRs and RTs. RTs to AV stimuli were significantly faster than those to An stimuli, while HRs to AV stimuli were significantly higher than those to both An and Vn stimuli. The behavioral facilitation of RTs and HRs might indicate that congruent semantic information within AV stimuli facilitated cross-modal integration and further improved the performance of the semantic discrimination task [31].

### The cortical network related to audiovisual semantic integration with attention

By implementing the (AV+Fn) > (An+Vn) contrast presented on the attended side, we obtained the cortical activation corresponding to the effect of semantic integration with attention (Fig 4(A)).

Two important regions of the TPJ and ATL were strongly activated during semantic integration with attention. These areas were widely discussed in multiple studies as association cortices of semantic representation [32–33]. The TPJ mainly contains the posterior temporal lobe and inferior parietal lobule. Compared to the unisensory auditory and visual inputs, AV stimulation induced stronger activation in the posterior temporal lobe, indicating that this region was involved in the semantic representation of cross-modal objects [34]. Similarly, Beauchamp *et al*. reported that the posterior temporal lobe showed an enhanced response when semantic auditory and visual object were presented together, relative to presentation in a single modality [12]. In addition, some studies demonstrated that the inferior parietal lobule, consisting of the angular gyrus and supramarginal gyrus, is an important region for cross-modal semantic representation [14, 35–36]. Geschwind and co-workers argued that the inferior parietal lobule is ideally connected as a cross-modal central region to code the semantic information contained in words [37]. In contrast, there has been an accumulation of functional neuroimaging [14, 38–39] studies of the ATL, which have found that the ATL is activated for a range of semantic tasks, irrespective of the modality of input (e.g., words, pictures, sounds, etc.) [40]. Moreover, temporary interference with ATL activity can produce semantic impairments across a range of tasks with words and pictures [41–42]. To further support these findings, conceptual impairments have been found in semantic dementia patients with ATL atrophy [43]. Here, TPJ and ATL were significantly activated, providing direct evidence that they are both involved in semantic integration. However, the role of these two regions as central hubs in cross-modal semantic representation has always been a matter of debate [14–18, 33, 44]. In the present study, the analysis of the nodal properties of the functional brain network showed that the two nodes with the highest degree and the highest nodal efficiency are the bilateral temporal pole (TP.L and TP.R) located in the ATL (Fig 5(B)). The high nodal efficiency reflects that the nodes connected to hubs are more tightly clustered together, and the high degree reflects that these nodes had an increased influence on general network processing [45]. The bilateral temporal pole regions showed the highest degree and nodal efficiency, indicating that these regions might contribute more to the information interaction efficiently. Moreover, the values of nodal degree and nodal efficiency decrease gradually from the anterior to posterior temporal lobe (Fig 5(B)), and the pattern of functional connectivity between bilateral TP and other regions within the temporal lobe showed that the connectivity strength gradually increased from posterior, middle, to the anterior areas, with the connectivity between the left and right temporal poles being the strongest (Fig 5(C)). Supporting our findings, neuroscience studies have indicated that convergence of sensory information in the temporal lobe is a graded process that occurs along both its longitudinal and lateral axes and culminates in the most rostral limits [46–47]. Thus, we suggested that both TPJ and ATL are important sites for semantic integration with attention, but ATL may serve as a central hub role in the cortical network.

Another significant activation related to semantic integration in the attention condition was found in the frontal and parietal cortices. The frontal cortex was demonstrated to belong to wide cortical networks representing cross-modal semantic associations [48–50], which receives afferent connections from multiple association areas, and integrates cross-modal information in support of behavior [48–50]. For example, an fMRI study showed that the frontal cortex was activated more strongly during semantically congruent compared to incongruent AV stimulation [49]. Naumer *et al*. found that multisensory stimuli of sounds and images with semantic information was integrated in the frontal cortex [50], suggesting that the frontal cortex is responsible for semantic information integration. In addition, FP, FOrb and MedFC in frontal cortex are the hubs with high nodal degree and nodal efficiency (Fig 5(B)), reflecting that frontal cortex contributes more to effective semantic integration with attention. Beyond the frontal cortex, the parietal cortex was also found to be similarly activated. Some studies have suggested that multisensory information would be integrated and represented in the frontal lobe via the parietal lobe [51–52], which is generally thought of as a higher-order association area and was observed to be activated in semantically congruent audiovisual integration [53]. These studies supported our finding of the activation in the frontal and parietal lobes during semantic integration, reflecting that these areas are both engaged in cross-modal semantic representation.

### The cortical network related to audiovisual semantic integration without attention

By implementing the (AV+Fn) > (An+Vn) contrast presented on the unattended side, we observed that the STG, HG and precentral gyrus were strongly activated (Fig 4(B)). Some ERP studies have suggested that cross-modal semantic processing occurs even when the semantic information is not actively attended [54]. Our present result supports this contention and further identifies the cortical regions responsible for semantic integration without attention.

The specificity of HG for semantic integration under the unattended condition was an interesting finding. This primary auditory cortex was activated in semantic integration with attention but not activated without attention, indicating that it may play an important role in unattended semantic integration. Neurophysiological studies in human and nonhuman primates have found early integration in the primary auditory cortex [55], and suggested that multisensory integration processes in the primary sensory cortices are governed by tight temporal [56] and spatial constraints [57]. However, in the present study, we removed the effect of early integration induced by a congruent temporospatial relationship through the experimental design with bimodal stimuli, and focused on the effect of semantic integration. Thus, our result showed that higher semantic integration occurred in the primary auditory cortex, suggesting that the multisensory integration in the primary auditory cortex is also related to a congruent semantic relationship.

Multisensory interactions in the primary auditory cortex can be mediated by several types of functional neural architectures, including feedforward thalamocortical, direct connections between sensory areas and feedback from higher-order association areas [58–59]. We suggested that one possibility is that the semantic information from the auditory and visual modalities was input into the HG from subcortical architectures, and then was integrated. Alternatively, the auditory and visual signals were combined in the association areas, such as the posterior STG here, and the subsequent outcome affected the response amplification in HG by means of feedback projections [60].

Our results indicated that HG is an important region for cross-modal semantic representation in the absence of attention. Another significant activation was noted in the precentral gyrus, which was suggested to be associated with decision-making of movement [52]. We speculated that this may reflect the mechanism of cognitive control, which inhibited the participants from responding to the stimuli on the unattended side by pressing a button. However, further studies are needed to confirm and elucidate these findings.

### The modulating effect of attention on semantic integration

By comparing the results of semantic integration with and without attention, we observed that in comparison with the unattended condition, when attention was allocated and directed to the stimuli, the integration sites were widely distributed in the temporal, frontal, and parietal lobes, indicating that attention strongly affected semantic integration processing [61–62]. First, attention modulated the response in the frontoparietal cortices, which were strongly activated in semantic integration with attention but were barely activated in integration without attention. This result supported the theory that frontoparietal cortex is a source of top-down attention [61], adjusting the neuron responses to favor information that is currently relevant for behavior [62]. This selective representation may serve as a source of bias, prioritizing the processing of task-relevant information across the brain [63]. We speculated that the allocation of attention resource enabled the neurons in the frontoparietal cortex to focus on processing the task-related stimuli presented on the attended side. Secondly, attention modulated the cross-modal semantic representation in the temporal cortex, which enhanced the response in ATL but inhibited the response in HG. The ATL as a central hub was specific to semantic integration with attention, which was not activated in the unattended condition, and the HG was specific to semantic integration without attention, which was not observed to be activated in the attended condition. The posterior STG was activated both in attended and unattended conditions, indicating that this region may be a generally site for semantic integration. These differences might indicate that ATL is responsible for conscious semantic integration that needs allocation of attention. In other words, attention is a prerequisite for semantic integration in ATL. In contrast, the semantic integration in HG did not require attention to be allocated, which might reflect an unconscious or a pre-attentive processing [26, 64]. We suggest that the activation patterns of cortical networks indicated that semantic integration is a complex cognitive process, which may contain multiple integration processes occurring in different cortical regions and are modulated by attention. However, further experimentation is required to clarify our speculation.

## Conclusion

In the present study, we explored the cortical networks underlying semantic integration and the modulation of attention by using fMRI and graph-based methods. The results showed that the cortical network related to semantic integration with attention was distributed in the frontal, parietal, and temporal lobes, while the cortical activation related to semantic integration without attention was located in the posterior STG, HG, and precentral gyrus. The different responses in cortical regions reflected that semantic integration could occur both in attended and unattended conditions, and attention can facilitate semantic integration to enhance cross-modal semantic representation [61–62]. We suggest that semantic integration with attention is a conscious process and needs a wide cortical network working together, in which ATL plays the role of a central hub. Semantic integration without attention is a pre-attentive process and involves a relatively small cortical network, in which the HG may play an important role. However, one limitation of our study is that our sample size was relatively small. It is important to validate our findings by replicating our analyses in a larger sample of subjects. Our present study would be useful to investigate multisensory integration and attention at multiple processing stages and levels within the cortical hierarchy.
